# Rivaroxaban Experience at Tertiary Care Centre in Saudi Arabia:A Retrospective Observational Study

**DOI:** 10.31661/gmj.v9i0.1882

**Published:** 2020-12-26

**Authors:** Muhammad Riazuddin, Mohamed Ahmed Mpharm, Muhammad Imran Butt, Mohammed Abufarhaneh, Shahid Masud Khan, Omar Emadi, Mohammed Saad AlKasab, Haifa Fahad Alsudairy, Khalid AlShammari, Aamir Sheikh, Abdulaziz AlRashed

**Affiliations:** ^1^King Faisal Specialist Hospital & Research Centre, Riyadh, Saudi Arabia

**Keywords:** Anticoagulants, Atrial Fibrillation, Rivaroxaban, Stroke, Thromboembolism

## Abstract

**Background:**

Atrial fibrillation (AF) and Venous Thromboembolism (VTE) are significant causes of morbidity and mortality. Direct oral anticoagulants (DOACs) are as effective as vitamin K antagonists (VKAs) with a propensity to cause less major bleeding. This study aimed to assess the safety and effectiveness of rivaroxaban in routine clinical practice in a large tertiary referral center in Saudi Arabia.

**Materials and Methods:**

In this study, patients who received rivaroxaban at a tertiary referral hospital were included in this study. All adverse events were recorded, including major bleeding, non-major bleeding events, symptomatic thromboembolic events (deep vein thrombosis, pulmonary embolism) and all-cause death.

**Results:**

A total of 509 patients were prescribed rivaroxaban during the study period. The most common indication for rivaroxaban was non-valvular AF (65.3%) and VTE (34.7%). The mean age was 60.4 ± 16.4 years, and 50.8% were female. The median CHA2DS2-Vasc score was 2.1 in patients on rivaroxaban for non-valvular AF. Bleeding occurred in 72 (14.1%) patients, of which 20 (3.9%) had major bleeds. Thrombosis events occurred in 13 (2.5%) patients in the overall cohort. Fourteen (2.7%) patients died during the study, including a case of fatal bleeding secondary to rivaroxaban.

**Conclusion:**

This study describes the use of rivaroxaban in a broad patient population in clinical practice in the Middle East. The overall bleeding and thrombosis rates in this study were comparable to those seen in major clinical trials.

## Introduction

There are few published studies available describing the prevalence of atrial fibrillation (AF) outside of Europe and North America [1]. Patients with AF in the Middle East tend to be younger with a higher prevalence of obesity, diabetes as well as higher rates of stroke and death [2]. There is also evidence that in Saudi Arabia, hospitalized patients do not receive adequate venous thromboembolism (VTE) prophylaxis, and consequently, there may be an underestimation of the mortality rates associated with VTE [3]. Anticoagulants can mitigate the risk of stroke in patients with AF and significantly reduce VTE recurrence in patients with a history of VTEs [4-6]. Vitamin K antagonists (VKAs) have, for decades, been the anticoagulation treatment of choice for patients with AF and VTE. The availability, in recent years of direct oral anticoagulants (DOACs) has offered a new modality of anticoagulation treatment for patients with AF and VTE. DOACs, such as dabigatran, apixaban, rivaroxaban, and edoxaban, have been shown to be as effective as VKAs in preventing stroke and VTE recurrence, a lower propensity to cause significant bleeds, particularly intracranial hemorrhage [7-13]. One of the first available DOAC was rivaroxaban a direct factor inhibitor factor Xa inhibitor, approved for the treatment and prevention of deep vein thrombosis (DVT) and pulmonary embolism (PE), as well as the prevention of stroke in patients with non-valvular AF. Rivaroxaban was first introduced at our institution, a tertiary referral hospital in Riyadh, Saudi Arabia, in 2011. In this study, we describe the safety and effectiveness of rivaroxaban in patients with VTEs and/or AF treated at our institution.

## Materials and Methods

This retroprospective study was conducted in a tertiary referral hospital in Riyadh, Saudi Arabia. Patients prescribed rivaroxaban between January 2012 and December 2015 were identified from the pharmacy prescribing system and subsequently included if they met the following inclusion criteria; a documented diagnosis of AF and/or VTE (DVT and/or PE) and were ≥18years of age. The exclusion criteria were the use of rivaroxaban for VTE prophylaxis post hip or knee replacement and patients with valvular AF (defined as the presence of rheumatic heart disease, heart valve replacement and mitral stenosis). The primary outcome was the safety of rivaroxaban as it relates to major bleeding. Major bleeding was defined using the International Society on Thrombosis and Haemostasis (ISTH) criteria [14]. ISTH major bleeding in non-surgical patients was defined as having symptomatic presentation and:

 1. Fatal bleeding, and/or

 2. Bleeding in a critical area or organ, such as intracranial, intraspinal, intraocular, retroperitoneal, intra-articular or pericardial, or intramuscular with compartment syndrome, and/or

3. Bleeding causing a fall in hemoglobin level of 20 g L−1 (1.24 mmol L−1) or more or leading to transfusion of two or more units of whole blood or red cells. Secondary outcomes included symptomatic thromboembolic events (DVT and PE), stroke/transient ischemic attack (TIA), death from any cause and clinically relevant non-major bleeding, defined as any sign or symptom of hemorrhage (e.g., more bleeding than would be expected for a clinical circumstance, including bleeding found by imaging alone) that does not fit the criteria for the ISTH definition of major bleeding, but did meet at least one of the following criteria; medical intervention by a healthcare professional, hospitalization or increased level of care to the patient face to face (i.e., not just a telephone or electronic communication) evaluation [15].

###  Statistical Analysis

 The analyses of events were descriptive and generally limited to frequency tables or summary statistics (e.g., mean+standard deviation [SD] or median+quartiles). Also. Independent sample t-test was performed; a P-value less than 0.05 was considered as significant differences.

###  Ethical Consideration

 The study was conducted in accordance with the Declaration of Helsinki and approved by the hospital research and ethics committee. Since this study was non-interventional and retrospective in nature and events retrieved from patients’ charts, patient consent was not required.

## Results

 A total of 509 patients were prescribed rivaroxaban during the study period. The baseline demographics and clinical characteristics are summarized in Table-1. The mean age was 60.4±16.4 years, and 50.8% were female; the most common indication for rivaroxaban was non-valvular AF (65.3%), followed by VTE (34.7%). For the evaluable patients (489), 17.5% had renal impairment (estimated glomerular filtration rate [eGFR]<60ml/min/1.73m2). The median CHA2DS2-Vasc score was 2.1 in patients treated with rivaroxaban for non-valvular AF. The most frequent comorbidities were hypertension 58.1%, diabetes 44.8% and congestive heart failure 12.1%. There was a history of stroke, TIA or systemic embolism in 42.9% of patients evaluated. There were 72 incidents of bleeding throughout the study period. A total of 20 patients (3.9%) experienced major bleeds as per the ISTH definition (Table-2). The most common sites of bleeding in patients with major bleeds were gastrointestinal (11 cases) and genitourinary (5 cases). Patients with renal impairment (eGFR<60ml/min/1.73m2) had a higher incidence of bleeding compared with those with normal renal function, 24% vs 12.5% (P <0.05). Patients with malignancy had a significantly higher incidence of bleeding than those without malignancy, 24.5% vs. 13% (P<0.05). Overall, 13 (2.5%) patients experienced thromboembolic events (Table-2). There were eight patients with stroke/TIA and four patients with VTEs. Patients with renal impairment (eGFR<60ml/min/1.73m2) had a numerically higher incidence of thrombosis compared to those with normal renal function, 10.7% vs. 5.3%; however, this did not reach statistical significance (P:0.3815). Patients with malignancy also had a numerically higher incidence of thrombosis, 4% vs. 2.4%; however, this was not statistically significant (P:0.3611). Fourteen (2.8%) patients died during the study period, with a single case of fatal bleed attributed to rivaroxaban.

## Discussion

In this retrospective observational cohort study, we describe the real-world use of rivaroxaban in a broad patient population treated at a large tertiary referral hospital in Saudi Arabia. The study population was relatively young, with a mean age of 60.4 years; this is consistent with other research, in which the age of patients treated with anticoagulation in the Middle East tend to be younger than those in the United States and Europe [2,3,16-17]. There was a high prevalence of comorbidities in this study, with the most common were hypertension (58.1%), diabetes (44.8%), and congestive heart failure (12.1%). Furthermore, a significant number of patients, 42.9%, had a history of stroke/TIA or systemic embolism. Most of the patients were prescribed rivaroxaban to treat non-valvular AF (65.3%), followed by VTE (34.7%). Nearly a fifth of patients had renal impairment, defined as eGFR<60mL/min/1.73m2. The overall rates of major or life-threatening bleeding and thrombosis in this study were low. Fatal and critical organ bleeding events, including intracranial hemorrhage, were rare, occurring in ≤1% of patients. The most common site of major bleeds was the gastrointestinal tract, and stroke was the most common form of thrombosis. The overall mortality rate during the study period was 2.8%. As expected, patients with renal impairment and malignancies had a higher incidence of bleeding. While thrombosis events were numerically higher in patients with malignancies and renal impairment than the overall cohort, this did not reach statistical significance. Incidence rates of major outcomes in this study were similar to those reported in other real-world studies that have assessed rivaroxaban [18]. For instance, the rates of bleeding and systemic embolism or stroke in the XANTUS (Xarelto for Prevention of Stroke in Patients with Atrial Fibrillation) trial were 1.7/100 and 1/100 patient-years, respectively. Key limitations of this study were its retrospective and observational design as well as the fact that it was conducted at a single institution. Consequently, it may not be possible to generalise the findings of this study. Another limitation was the potential for missing data, since all data was collected from patients’ electronic medical records.

## Conclusion

 This was one of the first studies assessing the safety and effectiveness of rivaroxaban in a broad patient population in the Middle East. The event rates among patients were broadly similar to those seen in other observational studies.

## Acknowledgment

 We are grateful to Amjad Alfifi and Edward Devol, at Department of Biostatistics, Epidemiology &Scientific Computing King Faisal Specialist Hospital & Research Center, Riyadh, Saudi Arabia

## Conflict of Interest

 The authors have declared no conflict of interest.

**Table 1:** Baseline Demographics and Clinical Characteristics

**Patients characteristics **	**Rivaroxaban (N 509)**
**Age (years), mean (± SD)**	60.4 (± 16.4)
**Gender (female), %**	50.8
**Weight (Kg), mean (± SD)**	82.8 (± 21.3)
**BMI (Kg/m** ^2^ **), mean (± SD)**	31.2 (± 7.59)
**Indication for rivaroxaban, (%)**
Non-Valvular AF	65.3
Venous thromboembolism	34.7
**Existing comorbidities, %**
Hypertension	58.9
Diabetes Mellitus	44.9
Prior stroke/non-CNS SE/TIA	42.9
MI/IHD	22.3
Antiphospholipid syndrome/SLE/ Major thrombophilia disorder	10
Malignancy	9.7
CHA_2_DS_2_-VASC score, median	2.1 (2.1)
Patients with evaluable eGFR, (mL/min/1.73m^2^), n	489
eGFR, 60<mL/min/1.73m^2^, %	17.5

* eGFR calculated using the modified MDRD Formula. **AF**: Atrial fibrillation; **BMI**: Body mass index; **CNS**: Central nervous system; **eGFR** Estimated glomerular filtration rate; **IHD**: Ischemic heart disease; MI: Myocardial infarction; **SD**: Standard deviation; **SE**: Systemic embolism; **SLE**: Systemic lupus erythematosus; **TIA**: Transient ischemic attack; **VTE**: Venous thromboembolism

**Table 2:** Incidence of Bleeding, Thromboembolic Events and All Causes of Death

**Outcomes**	**Incidence proportion** **n (%)**
**Total bleeds**	72 (14.1)
**Major bleeding, according to ISTH criteria**	20 (3.9)
**Fatal bleeding**	1 (0.2)
**Site of major bleed**
Intracranial hemorrhage	1 (0.2)
Critical organ bleeding^≠^	3 (0.6)
Gastrointestinal	11 (2.2)
Genitourinary bleeding	5 (1)
**Systemic thromboembolism**
Stroke/TIA, DVT & PE	13 (2.5)
**Site of systemic thromboembolism**
Stroke/TIA	8 (1.6)
DVT/PE	4 (0.8)
Unknown	1 (0.2)
Death from any cause^¥^	14 (2.8)

≠ Critical organs include intraspinal, intraocular, retroperitoneal, intra-articular or pericardial, or intramuscular with compartment syndrome ¥ Includes one case of fatal bleeding due to rivaroxaban
